# Lower S-adenosylmethionine levels and DNA hypomethylation of placental growth factor (*PlGF*) in placental tissue of early-onset preeclampsia-complicated pregnancies

**DOI:** 10.1371/journal.pone.0226969

**Published:** 2019-12-30

**Authors:** Sandra G. Heil, Emilie M. Herzog, Pieter H. Griffioen, Bertrand van Zelst, Sten P. Willemsen, Yolanda B. de Rijke, Regine P. M. Steegers-Theunissen, Eric A. P. Steegers

**Affiliations:** 1 Erasmus MC University Medical Center, Rotterdam, The Netherlands, Department of Clinical Chemistry; 2 Erasmus MC University Medical Center, Rotterdam, The Netherlands, Department of Obstetrics and Gynaecology; 3 Erasmus MC University Medical Center, Rotterdam, The Netherlands, Department of Biostatistics; University Medical Center Utrecht, NETHERLANDS

## Abstract

**Introduction:**

The pathophysiology of preeclampsia is largely unknown. Serum placental induced growth factor (PlGF) levels are decreased during second trimester pregnancy. Aberrant DNA methylation is suggested to be involved in the etiology of preeclampsia (PE). We hypothesize that DNA methylation is altered in PE placentas determined the methylation index by measuring placental S-adenosylmethionine (SAM) and S-adenosylhomocysteine (SAH) levels. In addition, we assessed global DNA methylation status by long-interspersed nuclear element-1 (*LINE*-1) and DNA methylation status of the *PlGF* gene.

**Methods:**

Placental tissue of 11 early onset PE (EOPE), 11 late onset PE (LOPE) and 60 controls consisting of 25 uncomplicated controls 20 fetal growth restriction (FGR) and 15 preterm births (PTB) controls was collected from a nested case-control study of The Rotterdam Periconceptional Cohort. RNA and DNA was isolated from placental tissue and DNA was treated with sodium bisulfite. SAM and SAH levels were measured by LC-ESI-MS/MS. Methylation of *LINE-1* and *PlGF* genes was analyzed by Sequenom Epityper and. mRNA expression of *PlGF* was assessed with qPCR. Differences were assessed by analysis of covariance (ANCOVA) corrected for gestational age and birth weight.

**Results:**

Placental SAM levels were significantly lower in placental tissue of EOPE pregnancies compared to PTB controls (mean difference -240 ± 71.4 nmol/g protein, P = 0.01). *PlGF* DNA methylation was decreased in placental tissue of EOPE cases versus LOPE (mean difference -17.4 ± 5.1%, P = 0.01), uncomplicated controls (mean difference -23.4 ± 5.4%%, P <0.001), FGR controls (mean difference -17.9 ± 4.6%, P = 0.002) and PTB controls (mean difference -11.3 ± 3.8% P = 0.04). No significant differences were observed in SAH, SAM:SAH ratio, *LINE-1* DNA methylation and *PlGF* mRNA expression between groups.

**Discussion:**

The hypomethylation state of the placenta in EOPE, which is reflected by lower SAM and *PlGF* DNA hypomethylation underlines the possible role of placental DNA hypomethylation in the pathophysiology of EOPE, which needs further investigation.

## Introduction

Preeclampsia (PE) is one of the most severe maternal pregnancy complications worldwide and affects 2–8% of all pregnancies [1]. The pathophysiological mechanism is not fully understood and therapy is mostly aimed at reducing blood pressure rather than to cure the disease. The molecular mechanism is thought to involve defective invasion of the spiral arteries into the maternal blood stream, which leads to maternal endothelial dysfunction and concomitant high blood pressure [1, 2]. However, preeclampsia is a heterogeneous disorder: it can occur as early-onset (EOPE; ≤34 weeks of gestation) or late-onset (LOPE; >34 weeks of gestation) disease. Both phenotypes share common risk factors but differences also exists: EOPE is associated with more adverse (fetal) effects compared to LOPE [3].

Previously, we and others demonstrated a role of one-carbon metabolism in relation to PE [1, 4–7]. This metabolism donates methyl groups for cellular methylation reactions. S-adenosylmethionine (SAM) donates its methyl group to DNA after which S-adenosylhomocysteine (SAH) is formed. SAH can be hydrolyzed into homocysteine by SAH hydrolase by a reversible reaction. SAH is suggested to be a potent inhibitor of methylation reactions [8]. Elevated levels of plasma homocysteine, which is a sensitive marker of disturbed 1-carbon metabolism, has been shown to be associated with increased levels of SAH, decreased methylation index (SAM:SAH ratio) and decreased global DNA methylation (such as methylation of *LINE*-1 repetitive elements) and decreased methylation of imprinted genes [8–10]. Several studies demonstrated an imbalance in the release of soluble fms-like tyrosine kinase (sFlt-1) and placental growth factor (PIGF) into the maternal blood stream in preeclamptic women [11–13]. The presence of sFlt-1 competes with Flt-1 in the binding of PlGF and causes maternal endothelial dysfunction resulting in preeclampsia. During second trimester pregnancy elevated levels of sFLt-1 and decreased levels of PIGF have been observed in preeclamptic complicated pregnancies compared to normotensive pregnancies [11, 14, 15] resulting in an increased sFlt-1/PlGF ratio.

We hypothesized that DNA methylation is decreased in placental tissue of PE pregnancies and assessed SAM and SAH levels, global *LINE-1* DNA methylation and methylation of the *PlGF* gene in placental tissue of EOPE and LOPE pregnancies compared to uncomplicated controls, fetal growth restricted (FGR) controls and preterm birth (PTB) controls.

## Methods

### Placental material

Between June 2011 and June 2013 placental tissue was collected from selected patients for a nested case-control study of the Rotterdam Periconceptional Cohort (Predict study), an ongoing prospective tertiary hospital-based cohort study conducted at the Erasmus MC University Medical Center Rotterdam [16]. EOPE and LOPE cases were selected as cases and uncomplicated pregnancies were selected as controls. In addition, we oversampled the uncomplicated control group with placental tissue from fetal growth restricted (FGR) pregnancies and preterm births (PTB) as additional controls to reduce confounding by differences in gestational age and birth weight as described previously [17].

PE was defined according to the International Society for the Study of Hypertension in Pregnancy as gestational hypertension of at least 140/90 mmHg accompanied by an urine protein/creatinine ratio of ≥ 30 mg/mmol, arising de novo after the 20th week of gestation. EOPE and LOPE were defined as being PE diagnosed before and after 34 weeks of gestation, respectively [18]. Uncomplicated control pregnancies were defined as pregnancies without PE, gestational hypertension, FGR or PTB. FGR controls were selected based upon fetal weight below the 10^th^ percentile for gestational age. Birthweight percentiles were calculated according to the reference curves of the Dutch Perinatal Registry [19]. PTB controls were selected as spontaneous delivery between 22 and 37 weeks of gestation. Woman with multiple birth pregnancies or pregnancies complicated by fetal congenital malformations were excluded from this study. All women signed a written informed consent and the study protocol was approved by the medical ethical committee of the ErasmusMC (METC number 2004–227).

### Sample collection

Within 30 minutes after delivery of the placenta, samples of 0.5 cm^3^ were taken from the fetal side of the placental tissue at 4 different sites in a 3 cm radius around the umbilical cord insertion, after carefully removing the membranes and 2 mm of the top placental layer. Placental tissue from 4 different biopsies was quickly rinsed in PBS and was snap frozen in liquid nitrogen in Eppendorf tubes and was stored at -80° until further analysis. Placental tissue was grinded from 30 mg tissue of one placental biopsy and frozen placental powder was directly added to a cell lysis buffer and stored in Eppendorf tubes at -80 °C until further analysis. Subsequently, genomic DNA and RNA was extracted using the Allprep DNA/RNA isolation mini kit (Qiagen, Hilden, Germany), according to manufacturer’s instructions. Another aliquot of grinded placental tissue was mixed in an acidic saline solution (800μl saline, 80μl 1M Acetic Acid) for SAM, SAH and total protein measurement.

### Placental SAM and SAH measurements

S-adenosylmethionine (SAM) and S-adenosylhomocysteine (SAH) concentrations were determined in grinded placental tissue with an isotope dilution liquid chromatography tandem MS method (ID-LC-MS/MS) as adapted from Gellekink *et al* [20]. In short, the acidified placental extracts were neutralized with 1 M NH_3_ and subjected to a SPE sample clean-up. 10 μl of sample was then injected on a 50x2.1 mm HPLC Atlantis C_18_ column (Waters, Etten-Leur, the Netherlands) and eluted using a methanol gradient in 0.1% aqueous acetic acid. Detection was performed on a Quattro Premier XE mass spectrometer (Waters, Etten-Leur, the Netherlands). The concentration of total protein in the grinded placental extracts was determined on a Cobas 701 analyzer using the urine/CSF total protein test kit (Roche Diagnostics, Almere, the Netherlands). The amount of SAM and SAH was expressed as nmol/g protein.

### Quantification of LINE-1 and PlGF DNA methylation

Global DNA methylation was measured by *LINE*-1 repetitive elements (Genbank: X58075) as previously described by Wang et al [21]. Primers of *PlGF* gene were designed according to Genbank sequences NC_000014.9. The following primers were selected: L1-FOR: 5’-aggaagagagGTGTGAGGTGTTAGTGTGTTTTGTT -3’, L1-REV 5’ -cagtaatacgactcactatagggagaaggctATATCCCACACCTAACTCAAAAAAT -3’, *PlGF*-FOR 5’-aggaagagagGTTGGATTTTTGGATGTTTTTATTT -3’, and *PlGF*-REV 5’-cagtaatacgactcactatagggagaaggctCAAACAACAACTCCCTTCTAAAACT -3’,. DNA (500ng) was bisulfite treated with EZ DNA methylation kit (Zymo research, USA) according to the instructions of the manufacturer. Bisulfite treated DNA was dissolved in a Tris/0.1xEDTA buffer in a final concentration between 5 and 7 ng/μL. All bisulfite treated DNA samples were stored at -80°C. PCR was performed in quadruplicate within 30 days after bisulfite treatment and all PCR reactions were performed simultaneously. Methylated DNA and unmethylated DNA control (Zymo research, USA) were mixed in a 4:1 ratio as a positive control. In addition, genomic DNA isolated from blood leucocytes was used throughout the whole procedure as a positive control. Bisulfite treatment was performed for 20 samples each run and contained both the Zymo control as well as the blood sample control besides 18 placental samples. PCR was performed in a total volume of 12μl. Reaction mixture contained buffer 1x, dNTP (0.2mM), MgCl_2_ (1.5mM), forward/reverse primer (4 pmol), Amplitaq Gold (0.5 units, Life Technologies, Netherlands) and 2μl bisulfite treated DNA. PCR program followed a touch down procedure with an initial denaturation at 95° for 10 min. First 5 cycles of denaturation at 95° for 20 sec, annealing at 65° for 30 sec, elongation at 72° for 1 min. Second 5 cycles of denaturation at 95° for 20 sec, annealing at 58° for 30 sec, elongation at 72° for 1 min. Followed by 39 cycles of denaturation at 95° for 20 sec, annealing at 53° for 30 sec, elongation at 72° for 1 min and a final elongation at 72° for 3 min. 3μl of the PCR product was tested on gel. All PCR products were stored at -20°C until Sequenom analysis. Sequenom analysis (triplicate) was performed accordingly to the instructions of the manufacturer. All samples for *LINE*-1 measurement were analyzed on a single spectrochip, the same was done for *PlGF* measurement. Methylation percentage was calculated by MassARRAY Epityper Analyzer Software (Sequenom, USA).

### Validation of *LINE*-1 and *PlGF* methylation assays

*LINE*_1 amplicon detected 8 CpG sites of which CpG site 4 was omitted because of a silent signal and CpG_10 could not be measured because of low mass error. Mean percentage of methylation, standard deviation (SD), and coefficient of variation (CV) were calculated from triplicate measurements of each CpG site. CV had to be less than 10% to be able to measure *LINE-1* DNA methylation precisely. The *PlGF* amplicon consisted of 15 CpG sites of which 6 CpG sites were omitted due to low/high mass or silent signal. Of the 9 CpG sites that potentially could be quantified only CpG site 1 could be quantified, which is located in the 5’flanking region of the *PlGF* gene. Other 8 CpG sites had a methylation of less than 5%, which resulted in a CV>20%. Mean percentage of methylation, SD, and CV were calculated from triplicate measurements of each CpG site. Mean methylation of *PLGF* was lower than *LINE-1* methylation and for this reason we could not measure *PLGF* methylation with the same precision as *LINE*-1. We therefore used CV<20% as cut-off instead of CV<10%.

### *PlGF* mRNA quantification

RNA was isolated from 82 placental samples of PE-complicated pregnancies and controls from the same biopt as from which DNA was isolated and RNA quality was assessed with the 2100 Bioanalyzer and RNA 6000 Nano kit (Agilent Technologies, Germany). Of these samples, 8 recorded a RIN number <2 and were left out for further procedure. Of the remaining 74 samples two recorded a RIN = 7, all other samples showed a RIN >8 500 ng of RNA was used for a single RT reaction using the Iscript RT supermix kit (Bio-Rad, The Netherlands) in a total volume of 20 μL (both Oligo-dT and random priming). Incubation was done for 5 min at 25°C, 20 min at 46 °C and 1 min at 95°C. RT reactions were performed in series of 22 samples combined with one No-RT control reaction. Afterwards RT reaction samples were diluted 2.5 times with sterile water. Expression analysis was performed in a 96-well format on a Taqman 7500 fast analyzer (ThermoFisher Scientific, USA). The following taqman tests were used; *PlGF* (Hs00182176_m1); Eukaryotic 18S rRNA (Hs99999901_s1) and Beta actin (H99999903_m1). Taqman tests were performed with 2 μL cDNA, 10 μL Taqman fast advanced mastermix and 1 μL expression assay mix in a total volume of 20 μL. Fast Cycling was done for 2 min 50°C., 2 min 95°C and 40 cycles of 3 sec 95°C, 30 sec 60°C. As 18S RNA was highly expressed in placental tissue taqman cycling was reduced to 30 cycles. Samples were run in duplicate and were repeated if Ct difference between the duplicates was found to be >0.3 Ct value. Data was analyzed with Qbase+ (Biogazelle, Belgium). Of all cDNA samples a pooled control sample was made. This pooled sample was used for a 10-times dilution series on every plate. These dilution series were used for PCR efficiency correction. Efficiencies found for *PlGF*, *18S* and *ACTB* were 1.95, 1.95 and 1.91 respectively. Normalization was done with 18S and ACTB (M = 0.562). CNRQ values are calculated relatively to study average and were exported and used for further statistical analysis.

### Statistical analysis

Normality of the tested variables was checked manually by histrograms and was in addition analyzed by Shapiro-Wilk test. Means with SD were calculated from normal distributed variables and median with interquartile range (IQR) were presented for skewed variables. Differences in maternal and neonatal characteristics between the five groups were assessed using ANOVA for normal distributed variables and Kruskal-Wallis test for skewed variables. Differences in mode of delivery, fetal gender and birth weight percentage were calculated with Chi-square test.

Difference in methylation markers among the groups were assessed by analysis of variance (ANOVA). In addition, correction for differences in gestational age and birth weight were assessed by univariate analysis of covariance (ANCOVA) followed by Bonferoni post-hoc analysis. *LINE*-1 amplicon was analyzed as the mean of all CpGs per sample to assess methylation of the whole differentially methylated region (DMR). *PlGF* DNA methylation was skewed but analysis showed that transformation did not result in normality. However, as the Shapiro-Wilk test statistic of the log-transformed *PlGF* DNA methylation improved after log-transformation (0.942 versus 0.836, log_*PlGF* versus *PlGF*, respectively)) we used log-transformed *PlGF* DNA methylation in further analysis. P<0.05 was considered to be significantly. Bonferroni correction was applied to correct for multiple comparisons of independent methylation markers. We accounted for five independent multiple comparisons (SAM, SAH, SAM:SAH ratio, *LINE-1* methylation and *PlGF* methylation). P<0.01 was considered statistically significant after Bonferroni correction (i.e. P = 0.05/5). Correlation between methylation markers was assessed with Pearson correlation. All analysis were performed in IBM SPSS statistics 25.

## Results

### Baseline descriptives

Descriptives of the cohort are described in Table 1. Systolic- and diastolic blood pressure were significantly increased in women with EOPE and LOPE complicated pregnancies (Table 1). In addition, significant differences in birth weight and gestational age were present among EOPE, LOPE and the control groups (Table 1, ANOVA P<0.001).

**Table 1:** Maternal and neonatal characteristics.

	EOPE	LOPE	Uncomplicated controls	Normotensive FGR controls	Normotensive PTB controls	P value
	N = 11	N = 11	N = 25	N = 20	N = 15	
*Maternal*						
Age (y)	31.2 ± 4.0	33.6 ± 4.7	31.8 ± 5.3	29.6 ± 6.3	31.4 ± 3.3	0.30
SBD (mm Hg)	174 ± 19	150 ± 10	129 ± 12	130 ± 14	126 ± 17	<0.001
DBD (mm Hg)	99 ± 16	94 ± 11	76 ± 6	75 ± 9	79 ± 14	<0.001
Delivery (% vaginal)	1 (9%)	6 (55%)	18 (72%)	12 (60%)	12 (80%)	0.01
*Neonatal*						
Sex (male), N (%)	3 (27)	5 (45)	14 (56)	12 (60)	7 (47)	0.464
Gestational age (days)	215 (24)	265 (16)	279 (13)	267 (15)	244 (62)	<0.001
Birthweight (grams)	1181 ± 356	3363 ± 662	3788 ± 398	2451 ± 362	2259 ± 958	<0.001
Birth weight <10^th^, N (%)	11 (100)	1 (9)	0 (0)	20 (100)	0 (0)	<0.001

Data are presented as mean ± standard deviation or as ^median (IQR) for skewed variables for continuous variables and as number and percentage for dichotomous variables. EOPE = early onset PE; LOPE = late onset PE; FGR = fetal growth retardation; PTB = preterm birth; SBD = systolic blood pressure; DBD = diastolic blood pressure.

### Placental SAM and SAH levels

SAM levels were significantly different among the five groups (crude P = 0.002, Table 2). Post hoc analysis demonstrated significantly lower SAM levels in placental tissue from EOPE complicated pregnancies compared to PTB controls, which was independent from differences in gestational age and birthweight (mean difference -240 ± 71.4 nmol/g protein, P = 0.01, Table 2). However, this difference attenuated after Bonferoni correction as it did not reach the predefined Bonferoni significance level of P<0.01. SAH levels were not significantly different between EOPE, LOPE and each of the control groups (Table 2). SAM:SAH ratio was slightly lower in EOPE compared to PTB controls, but this was not statistically significant (mean difference -2.5 ± 0.99, P = 0.13). No differences were observed in SAM:SAH ratio between LOPE and each of the control groups (Table 2).

**Table 2:** Biomarkers in placental tissue of PE complicated pregnancies and controls.

	EOPE	LOPE	Controls	FGR controls	PTB controls	Crude P	Adjusted P
SAM (nmol/g protein),(n)	397 ± 109* (10)	345 ± 119 (10)	384 ± 147 (25)	360 ± 128 (20)	568 ± 241 (14)	0.002	0.02
SAH (nmol/g protein) (n)	95.8 ± 27.0 (10)	79.7 ± 29.0 (10)	78.5 ± 28.4 (25)	76.5 ± 31.8 (20)	84.1 ± 27.1 (14)	0.049	0.75
SAM:SAH ratio,(n)	4.4 ± 1.4 (10)	4.9 ± 2.4 (10)	5.3 ± 2.0 (25)	5.1 ± 2.3 (20)	6.8 ± 2.1 (14)	0.07	0.07
*LINE*-1 DMR methylation (%), (n)	22.1 ± 2.4 (11)	22.7 ± 1.9 (11)	22.8 ± 2.1 (25)	22.0 ± 2.5 (20)	22.3 ± 2.9 (14)	0.77	0.15
*PlGF* methylation (%)^$^, (n)	13.3 [3.7]^#^ (11)	13.0 [2.3] (11)	14.0 [2.8] (25)	12.2 [4.6] (20)	15.2 [7.6] (14)	0.15	0.001
*PlGF* mRNA^, (n)	1.19 ± 0.80 (10)	1.26 ± 0.69 (10)	1.21 ± 0.42 (21)	0.93 ± 0.54 (18)	1.22 ± 0.55 (14)	0.49	0.61

Data are presented as mean ± standard deviation or as ^$^median (IQR) for skewed variables. Crude P value of ANOVA test between 5 groups; Adjusted P value of ANCOVA corrected for gestational age and birthweight between 5 groups.

*P = 0.01 compared to PTB controls.

^#^P<0.05 compared to either LOPE, uncomplicated controls, FGR controls and PTB controls. EOPE = early onset PE; LOPE = late onset PE; FGR = fetal growth retardation; PTB = preterm birth; SAM = S-adenosylmethionine; SAH = S-adenosylhomocysteine; LINE-1 = long-interspersed nuclear element 1; PlGF = Placental growth factor.

^Calibrated normalized relative quantity as calculated with Qbase+

### Placental LINE-1 and PlGF DNA methylation

*LINE*-1 DNA methylation was not significantly different in placental tissue among the groups (mean difference ranged from -0.009 till -0.02, P>0.05, Table 2). Placental *PlGF* DNA methylation was not significant among groups (mean difference ranged from -0.07 till 0.01, crude P = 0.15, Table 2). However, after correction for gestational age and birthweight *PlGF* DNA methylation was lower in EOPE compared to LOPE (mean difference -17.4 ± 5.1%, P = 0.01), uncomplicated controls (mean difference -23.4 ± 5.4%, P < 0.001), FGR controls (mean difference -17.9 ± 4.6%, P = 0.002) and PTB controls (mean difference -11.3 ± 3.8%, P = 0.04). The difference between EOPE compared to uncomplicated controls and FGR controls met the predefined Bonferoni level of P<0.01. Placental *PlGF* DNA methylation was not significantly lower in LOPE placentas compared to each of the control groups (Table 2).

### Placental PlGF mRNA levels

*PlGF* mRNA levels were not significantly different between EOPE, LOPE and each of the control groups (mean difference ranged from -0.01 till 0.43 after correction for gestational age and birth weight, P>0.05, Table 2). In addition, *PlGF* mRNA levels were modestly negatively correlated to *PlGF* DNA methylation in uncomplicated controls (R = -0.51, P = 0.02), whereas in EOPE, LOPE, FGR controls and PTB controls no significant correlation was present.

### Correlation of methylation markers

We investigated correlation of *PlGF* DNA methylation and SAM with other biomarkers. SAM was significantly correlated to SAH (r = 0.45, P = 0.001), SAM:SAH ratio (r = 0.51, P < 0.001), birth weight (r = -0.26, P = 0.02), and gestational age (r = -0.39, P = 0.006). In addition, *PlGF* DNA methylation was significantly correlated to SAM levels (R = 0.31, P = 0.006), SAM:SAH ratio (R = 0.24, P = 0.04), *LINE-1* DNA methylation (R = 0.40, P <0.001) and gestational age (R = -0.35, P = 0.02) although correlation coefficients were modest (R<0.80).

## Discussion

The pathophysiological mechanism of PE is unknown and studies have suggested a role of epigenetics. We show significantly lower SAM levels in placental tissues from EOPE complicated pregnancies compared to PTB controls. Concomitantly, *PlGF* DNA methylation levels were decreased in EOPE pregnancies compared to LOPE and each of the control groups. No significant differences were found in LOPE and neither was global *LINE-1* DNA methylation and *PlGF* mRNA expression significant different between EOPE, LOPE and each of the control groups.

Few studies have investigated DNA methylation at a global level and several studies focused at candidate genes [22], [23]. We and others provided evidence for a role of some novel epigenetic loci using methylation arrays although these results need to be validated in larger cohorts [24, 25]. None of the candidate studies have identified *PlGF* as target locus in PE neither was the *PlGF* locus identified in genome-wide methylation studies [26–29]. However, most studies consisted of only a few patients or did not correct for differences in confounders such as gestational age, which could have complicated the reported findings.

Based on the previous described role of altered one-carbon metabolism in relation to PE we hypothesized that changes in SAM, SAH and SAM:SAH ratio are present in PE, which lead to decreased methylation. We showed significantly lower SAM levels in placental tissue of EOPE pregnancies compared to PTB controls, which result in a lower SAM:SAH ratio, although this ratio was not significant (P = 0.13). These lack of significant lower SAM:SAH ratio might be caused by the relative small sample size of our study. SAM:SAH ratio has been described as the methylation potential of the cell and lower levels of SAM or are expected to be associated with less methylation. In addition, we did find a modest positive correlation of placental SAM levels with *PlGF* DNA methylation, which suggests that less methyl donor availability from SAM leads to decreased *PlGF* DNA methylation. More studies with larger sample size are necessary to evaluate the potential role of SAM in PE.

We were not able to demonstrate differences in placental *LINE-1* methylation between PE pregnancies and controls. Not many studies have investigated the role of global methylation measured by *LINE*-1 repeats in preeclampsia. Placental *LINE-1* DNA hypermethylation was found in two studies [30, 31] whereas another study found LINE-1 hypomethylation [23, 32]. In three other studies, placental *LINE*-1 methylation was not significantly changed in PE compared to controls [33–35], which is in agreement with our results that *LINE*-1 methylation in placental tissue of PE complicated pregnancies is not different from control pregnancies. However, more studies are necessary to draw a firm conclusion.

In our study we assessed placental methylation status of *PlGF* in PE complicated pregnancies and compared this to uncomplicated pregnancies and pregnancies complicated by FGR and PTB. In most studies, DNA methylation of PE complicated pregnancies was compared to uncomplicated controls. However, uncomplicated controls differ significantly from PE complicated pregnancies in gestational age and birth weight. To circumvent this we included FGR-, and PTB complicated controls, which are more comparable in birth weight and gestational age to PE complicated pregnancies. However, despite including these control groups we still observed significant differences in birth weight and gestational age between PE complicated pregnancies and PTB- and FGR complicated controls for which we corrected. In addition, we found a modest negative correlation between *PlGF* DNA methylation and SAM levels with gestational age. In addition, SAM was also correlated to birth weight. These findings demonstrate that correction for birth weight and gestational age is needed to assess independency and also underlines that these methylation levels change during pregnancy, which warrant further studies during second trimester pregnancy.

We demonstrated *PlGF* DNA hypomethylation in placental tissue from EOPEpregnancies compared to each of the control groups, which was not present in LOPE. The underlying mechanism in EOPE and LOPE differs. It has been suggested that in EOPE poor placentation plays a dominant role, whereas in LOPE inflammatory processes are involved [1]. Studies have shown that PlGF levels are lower and have better diagnostic ability in EOPE than LOPE, which underlines our finding of *PlGF* DNA hypomethylation in EOPE but not in LOPE [36, 37].

To assess whether the observed lower *PlGF* DNA hypomethylation in EOPE is associated with lower *PlGF* mRNA expression we quantified mRNA expression in placental tissue but did not find evidence for changes in *PlGF* mRNA levels between EOPE, LOPE, and each of the control groups, which is in line with a previous study which showed that *PlGF* mRNA expression was not altered in EOPE placentas [38].

Interestingly, we did find that *PlGF* DNA methylation was negatively correlated to *PlGF* mRNA expression in uncomplicated controls, whereas in pregnancies complicated by EOPE, LOPE, FGR or PTB no significant correlation was observed. This suggests that under physiological circumstances *PlGF* mRNA expression might be regulated by *PlGF* methylation. However, under pathophysiological circumstances, such as EOPE, different regulation of PlGF methylation and mRNA expression occurs, which need to be further examined in future studies.

During second trimester pregnancies lower PlGF plasma levels have been observed in EOPE pregnancies. In our study *PlGF* DNA methylation was not associated with *PlGF* mRNA expression, which could have the following explanations; 1) *PlGF* promoter DNA methylation does lead to changes in *PlGF* mRNA expression, 2) due to large variation in *PlGF* mRNA expression we were not able to detect subtle differences in mRNA expression, 3) the physiological mechanism of *PlGF* downregulation at birth might differ from second trimester pregnancy. Future studies investigating *PlGF* DNA methylation in maternal plasma or chorionic villi obtained during second trimester pregnancy will be needed to assess whether the lower *PlGF* DNA methylation levels can be used to predict EOPE in the near future.

This is the first study that assessed placental DNA methylation of global markers of methylation and *PlGF* DNA methyalation in preeclampsia. We showed lower SAM and *PlGF* DNA hypomethylation in placental tissue of EOPE complicated pregnancies compared to PTB controls. However as changes in methylation markers were modest more studies are needed to validate our findings and to assess the clinical value in relation to prediction of EOPE during first-trimester pregnancy.
